# Sensory disturbance along the inferior alveolar nerve as a first clinical sign of multiple florid cemento-osseous dysplasia of the mandible—A case report

**DOI:** 10.1016/j.ijscr.2018.11.036

**Published:** 2018-11-22

**Authors:** Pascal Grün, Patrick Bandura, Andrew Grün, Walter Sutter, Oliver Meller, Dritan Turhani

**Affiliations:** a Centre for Oral and Maxillofacial Surgery, University of Dental Medicine and Oral Health, Danube Private University, Steiner Landstraße 124, 3500, Krems-Stein, Austria; b Maxillofacial Private Surgery, Rottenburg am Neckar, Germany

**Keywords:** FCOD, florid cemento-osseous dysplasia, NAI, nervus alveolaris inferior, CT, computed tomography, Case report, Florid cemento-osseous dysplasia, Sensory disturbance, Tumour excision, Cemento-osseous dysplasia, Osteomyelitis of the jawbone

## Abstract

**Introduction:**

Florid cemento-osseous dysplasia (FCOD) is a rare, benign, slow growing, multifocal fibro-osseous tumour originating from the periodontal ligament. The lesion is characterised by regular bone-structure replacement with fibrous tissue and dysplastic bone. The initial characteristics of FCOD, depending on localisation, resemble those of periapical lesions of inflammatory origin, potentially leading to misdiagnosis. In the absence of infection signs, the treatment of choice is conservative therapy with antibiotics and regular follow-up examinations.

**Presentation of case:**

We report the case of a 39-year-old Caucasian woman with sensory disturbance along the inferior alveolar nerve for >2 weeks. The involved teeth were vital and the digital orthopantomogram indicated multiple bilateral periapical brightening. Biopsy was indicated to exclude malignancy and to confirm the FCOD diagnosis with predominantly reactive calcification and cell-rich connective-tissue reaction. The sensory disturbances disappeared after the biopsy. Further conservative therapy included regular radiological examinations every 2 years to prevent surgical intervention due to infection risk.

**Discussion:**

Sensory disturbances around the nervus alveolaris inferior are well-known complications of dental and maxillofacial operations and of malignant tumours. Sensitivity disorder as a first symptom of fibro-osseous dysplasia has not been previously described. Although uncommon in its clinical behaviour, FCOD was finally diagnosed after thorough clinical, radiological, and histological examinations.

**Conclusion:**

Paraesthesia combined with FCOD has not been previously described. Correct diagnosis is essential for proper treatment and to avoid further complications even when the clinical symptoms appear unrelated.

## Highlights

• Florid cemento-osseous dysplasia (FCOD) is a rare, benign fibro-osseous tumour.
• We describe the case of a 39-year-old woman with FCOD and sensory paraesthesia.
• Biopsy was necessary for diagnosis to exclude malignancy and relieve the symptoms.
• We suggest that the paraesthesia resulted from nerve irritation caused by the FCOD.
• To our knowledge, this is the first report of paraesthesia combined with FCOD.

## 1 Introduction

Osseous dysplasia includes a large spectrum of non-neoplastic, fibro-osseous transformations. The current classification of bone-related lesions, launched in 2005 by the World Health Organization, does not account for the various phenotypes of fibro-osseous lesions [1].

The term florid cemento-osseous dysplasia (FCOD) was introduced in 1976 to describe an extensive variant of a reactive fibro-osseous process, known as cementoma [2]. FCOD is a rare, benign, multifocal fibro-osseous dysplastic process affecting tooth-bearing areas of the jaw, characterised by replacement of normal trabecular bone with osseous tissue and dense acellular cementum in a fibrous stroma [3]. Among the fibro-osseous lesions, cemento-osseous dysplasia is most commonly encountered in clinical practice [4]. It is characterised by replacement of normally-structured bone with fibrous, mineralised tissue [2,4] and can be divided into three subtypes according to its clinical and radiological features: periapical cemento-osseous dysplasia, focal cemento-osseous dysplasia, and FCOD [5].

In radiological examinations, the lesion manifests as multiple, lobular, well-marginated, non-expansile intraosseous masses of varying internal lucency and sclerosis, surrounding the root apices of vital teeth [3].

While all CODs share similar microscopic features, FCOD is distinguished by a multifocal distribution, involving two or more quadrants of the maxilla and mandible, often in a bilateral symmetric fashion. Female patients, specifically of African-American ethnicity, are more often affected than their male counterparts. The age at the time of diagnosis ranges from 19 to 76 years [6,7].

A systematic review found that in 59% of cases, the lesion occurred in Afro-Americans, in 37% of cases in Asians, and in 3% in Caucasians. Of all patients, 97% were female [8].

The origin is unknown, but currently, a reactive process is assumed. In most cases, the patients have no related heredity and no family history of similar lesions [9].

Because of the asymptomatic presentation of FCOD, the lesion can remain undetected for several years and is therefore often diagnosed accidentally during regular radiological examinations [10,11]. In severe cases, FCOD can result in bone expansion accompanied by pain and facial deformity [12]. FCOD must be distinguished from other dysplastic, neoplastic, and infectious processes of the jaw with overlapping radiological features [3] and is not associated with other skeletal abnormalities or biochemical markers. Combined with the absence of other systemic manifestations this contributes to the differential diagnosis [13].

Due to their rarity, histological diagnosis of fibro-osseous lesions is challenging, as they are easily confounded by similar tumors [3,7].

Unnecessary biopsy and surgery should be avoided, as the hypo-vascular lesions are susceptible to infection, osteomyelitis, and bone necrosis [14]. Accordingly, it is important to be aware of this differential diagnosis to prevent misdiagnosis, which would result in inappropriate treatment [3]. The gold standard treatment of asymptomatic, non-infected FCOD consists of regular/ routine radiographic follow up.

Sensory disturbances in the region of the nervus alveolaris inferior (NAI), including anaesthesia, paraesthesia, hypoaesthesia, and hyperaesthesia, are well known complications of malignant tumours and of dental and maxillofacial operations [15]. Infections and overfilled root-canal treatments can also irritate or disturb the nerve [16].

The aim of this case report is to highlight the clinical and radiographic features of FCOD to raise the general awareness for this rare disease. The patient was managed in a private dental office. We report the present case in accordance with the SCARE criteria [17].

## 2 Presentation of case

We report the case of a 39-year-old Caucasian woman who had persistent paraesthesia of the right NAI for longer than 2 weeks.

She had unremarkable drug, family, and psychosocial histories and was a non-smoker. Clinical examination revealed throbbing pain at the lingual and buccal sites of the mandible.

The orthopantomogram revealed multiple radiolucent, periapical lesions in different sizes and shapes. The findings were non-symmetrical, arranged bilaterally, and covered the entire lower jaw. Teeth 36 and 37 showed a large radiolucent periapical area, only separated via a small sclerotic septum. The origin of the lesion at tooth 36 appeared to be the distal radix and clearly expanded over to the apical of tooth 35. The canalis alveolaris inferior sinister appeared to be infiltrated at region 37. Further radiolucent lesions could be found periapically at region 33, almost reaching tooth 32. In the right mandible, similar lesions could be seen at regions 46, 42, and 41. Yet, the nerve channel appeared to be intact. The tumorous lesions to the front almost appeared as a single, large body. Although, every pathology arose around the radices, no root resorption was visible, and the performed vitality test was positive for all involved teeth. In contrast, no radiological pathologies could be found in the upper jaw.

**Fig. 1:**
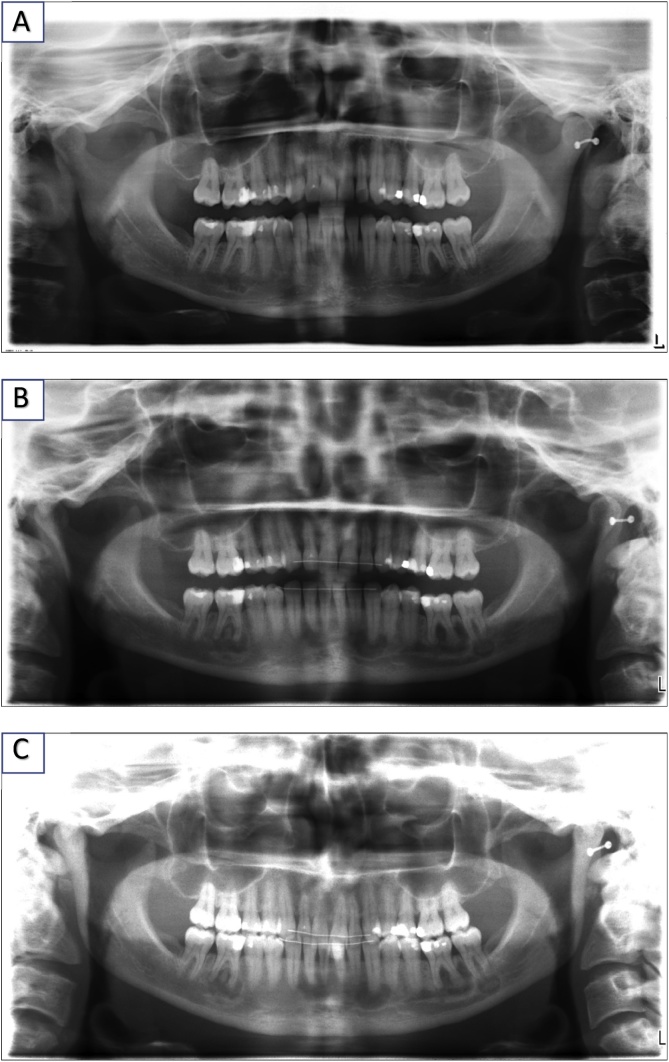
A: Panoramic radiograph acquired over 12 years before surgery. Note the elucidations at the first molars in the mandible. B. Panoramic radiograph one year before surgery. Note the increasing of the FCOD in region 36/46; 31; 42. C. Panoramic radiograph shortly before the operation.

On an orthopantomogram obtained 13 years earlier, some of the radiolucent lesions of the lower jaw could already be assumed at regions 37, 36, 32, and 46 (Fig. 1 (A–C)).

**Fig. 2:**
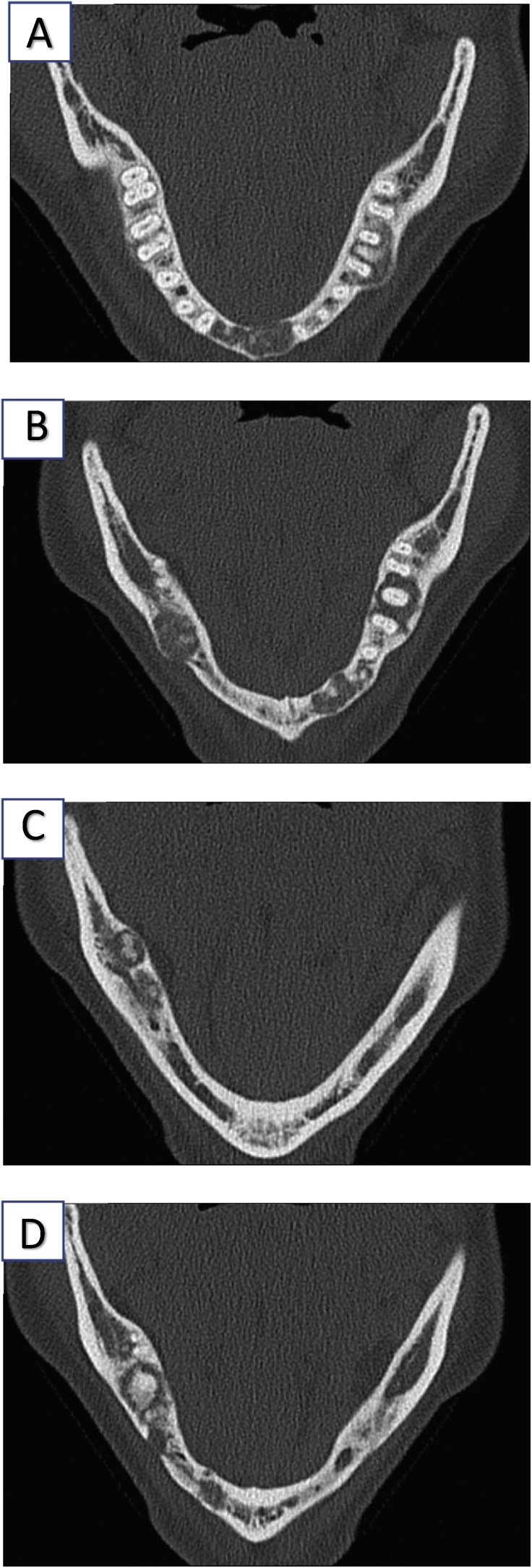
A–D are the computed tomography images, which show the successive layers of the computed tomography in the horizontal cut.

**Fig. 3:**
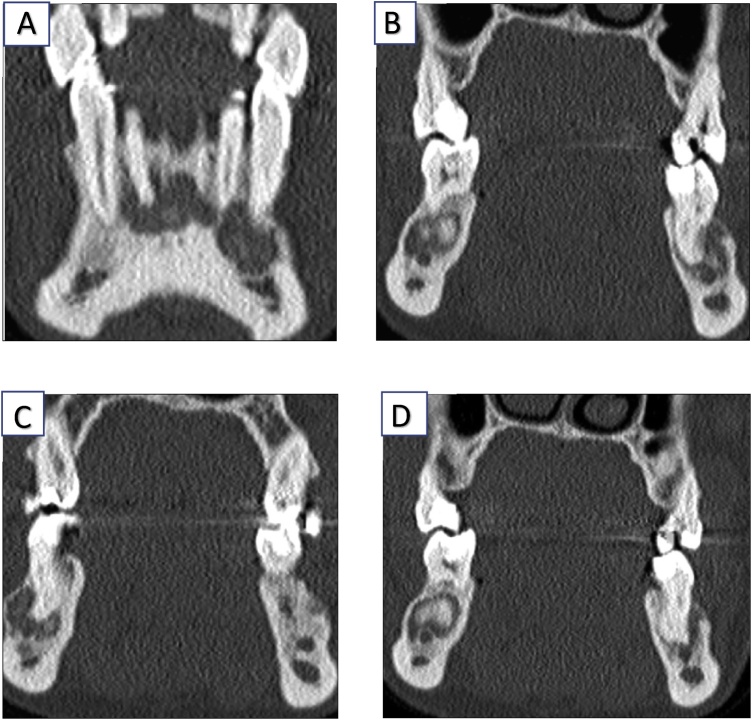
A–D are the computed tomography images, which shows us different layers of the CT in the horizontal cut.

Computed tomography (CT) of the mandible 13 years after the first CT, a high-resolution 1-mm stratification of the horizontal mandibular branch, showed multiple resorption zones periapical directly in the area of incisors 31, 41, and 42. In addition, periapically, osteolysis was observed in the area of premolar 35 and molars 36, 37, 46, and 47. The corticalis was thinned and displaced ventrally, whereas the alveolar canal had remained intact. The lesion was more distinct on the left side than on the right. There was no periosteal reaction and no spicules Fig. 2 (A–D) and Fig. 3(A–D).

Based on the medical history, clinical symptoms, and radiological findings, a differential diagnosis of fibro-osseous disease was decided.

However, due to the existing paraesthesia in the area of the left NAI, a histopathological examination was necessary to exclude malignancy. The specimens were acquired from the area around region 46.

**Fig. 4:**
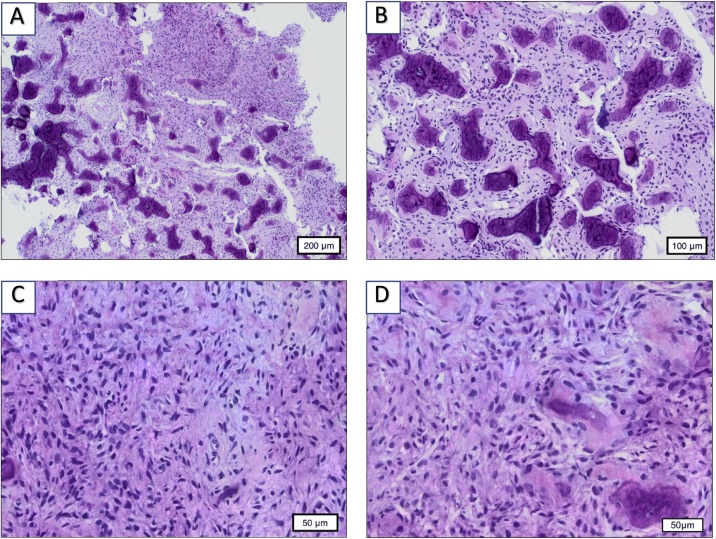
A–D. The histological findings in different enlargements (200 μm; 100 μm; 50 μm). It shows the mixture of bone and connective tissue.

A combination of surgical bur and the chisel technique was used for bone removal. First, the surgical bur was cut under water cooling conditions and then the bone was removed with the chisel technique. The semi-lunar flap was selected as the ideal access path for the sampling Fig. 4(A–D).

**Fig. 5:**
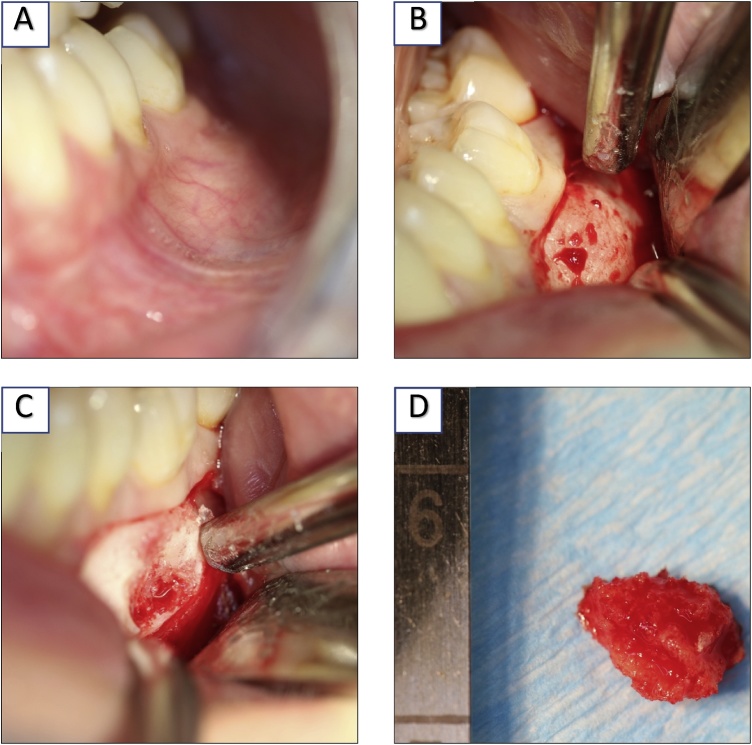
A. Buccal intraoperative view before operation. B. Intraoperative view after surgical opening. C. OP-situation after bone biopsy D. The removed bone biopsy.

The histological examination revealed calcifications or map-type neoplasms in front of a relatively cell-rich, fibrous stroma, with a small number of histocytic cell elements. The histological appearance was compatible with the diagnosis of FCOD. Typical of FCOD, as in our case, the teeth were vital despite the apical localisation of the lesions Fig. 5(A–D).

After the biopsy, the paraesthesia of the bottom lip disappeared. Six months later, the patient described a feeling of intense painful pressure in the area of the former FCOD. Although uncommon and aware of the possible risks, we decided to perform a surgical procedure. The patient underwent surgery under general anaesthesia performed by the head of the dental department. Using an intraoral approach, a mucoperiosteal flap was raised. The aim was to partially remove the tumour, especially at the area of the nervus mandibularis, which we suspected was the origin of the symptoms. At that time, the patient also received perioperative antibiotics. However, no evidence of FCOD could be detected upon specimen examination. The specimen appeared as regular modelled bone with physiological circulation. The patient received postoperative antibiotics, a nonsterial anti-inflammatory agent, and a proton pump inhibitor, and was discharged in stable cardiorespiratory condition. For the following 6 months, the patient remained under regular follow up. Neither further increase in size of the lesion nor newly occurred spots were detected.

Biopsy and histopathological examination confirmed the suspected diagnosis. Due to the successful operation, with no postoperative complaints and uneventful wound healing, the patient was very satisfied. The therapy was very well tolerated, and the patient reported no pain.

## 3 Discussion

We presented the case of a young Caucasian woman with large FCOD affecting the mandible. Our patient and her family did not have hereditary diseases. Further, there has been no documented case of familial FCOD [9].

FCOD is normally described as a symmetrically-arranged lesion affecting the left and right part of the lower jaw [6,7]. However, this symmetry could not be seen in our case; a rarely described finding.

Further we can confirm that FCOD radiologically appears as a well-defined lesion that includes the apices of vital teeth and can spread across multiple quadrants [3], as for example in our case, across multiple parts of the mandible. As previously described, FCOD remained asymptomatic for a long time in our patient [3,10,11]. FCOD is usually discovered by chance [1,11]. This differs from our case, where the patient presented with sensory disorder as a first sign of the pathology.

To our knowledge, sensory disturbances, as a first sign of FCOD, have not been described in the literature [18]. The medical history of the patient was inconspicuous. Because she did not require endodontic treatment or any other surgical procedure, we suspect that the nerve was irritated due to the close and partially infiltrating tumour. Our hypothesis was confirmed, as the paraesthesia and the dull feeling of pressure disappeared after the surgery.

We were very well aware of the fact that an invasive treatment could trigger an infection in the hypo-vascular lesion, but due to the paraesthesia, a biopsy was essential to exclude malignancy.

Further therapy includes radiograph examinations every 2 years and surgical procedures should be avoided due to the risk of infection [19]. We believe that the best therapy for our patient is conservative symptom alleviation and observation. Earlier detection would not have resulted in other therapy and the disease course would not have been altered. Preventive intervention may therefore not be desirable, as it may cause later complications. An early diagnosis would have had the advantage that we could have more accurately recognised the disease course and the growth change of the dysplasia. Sensory disturbances can be versatile and are often considered unpleasant; they can be caused by harmless irritation of the nerve or a serious condition of the nervous system. For this reason, the cause had to be followed up. Luckily, in this case, the cause was irritation of the nerves that was untypically triggered by the FCOD.

In principle, surgery is strictly contraindicated, but in our case of a sensory disorder, it was useful to eliminate malignancy and relieve pain and remove pressure. If the size of the lesion increases, a modelling osteotomy will have to be considered.

## 4 Conclusion

This report describes a case of FCOD, a rare and benign pathology. Unlike its normal clinical behaviour, the patient in our case had symptomatic paraesthesia in the mandible as a first sign of the lesion. To the best of our knowledge, this is the only documented case of such symptomatic FCOD in the literature. The growth of the lesion could be documented for more than 13 years. We assume that the expanding lesion triggered the sensory disorder in the mandible when it reached the nerve. Sensory disturbances in cases of FCOD are not common and by the time they appear, they are usually due to infection of the lesion or iatrogenic injury during an operation or an endodontic treatment; none of which applied in our case. Another particularity is the slightly asymmetric growth of the lesion and the ethnicity of our patient. It is our concern that every radiolucent apical lesion on an orthopantomogram should be carefully considered for a more infrequent differential diagnosis. Therefore, a vitality test should be performed before any further treatment is undertaken to avoid misdiagnosis and unnecessary interventions.

## Conflicts of interest

The authors declare that there is no conflict of interest.

## Funding

This research received no specific grant from any funding agency in the public, commercial, or not-for-profit sectors.

## Ethical approval

The ethical approval has been exempted by our institution.

## Consent

The patient received a thorough explanation of this report gave her oral and written informed consent to be included in this report as well as for publication of these case, anonymous data, and pictures. A copy of the written consent is available for review on request.

## Author contribution

Pascal Grün and Patrick Bandura: study concept and design, writing the paper.

Andrew Grün, Walter Sutter and Oliver Meller: data collection, analysis and discussion of data.

Dritan Turhani: final approval of the version to be published.

## Registration of research studies

Not applicable.

## Guarantor

The corresponding author is the guarantor of submission.

## Provenance and peer review

Not commissioned, externally peer reviewed.
